# A Dynamic Recurrent Neural Network for Predicting Higher Heating Value of Biomass

**DOI:** 10.3390/ijms24065780

**Published:** 2023-03-17

**Authors:** Babak Aghel, Salah I. Yahya, Abbas Rezaei, Falah Alobaid

**Affiliations:** 1 Institut Energiesysteme und Energietechnik, Technische Universität Darmstadt, Otto-Berndt-Straße 2, 64287 Darmstadt, Germany; 2 Department of Chemical Engineering, Faculty of Energy, Kermanshah University of Technology, Kermanshah 6715685420, Iran; 3 Department of Communication and Computer Engineering, Cihan University-Erbil, Erbil 44001, Kurdistan Region, Iraq; 4 Department of Software Engineering, Faculty of Engineering, Koya University, Koya KOY45, Kurdistan Region, Iraq; 5 Department of Electrical Engineering, Kermanshah University of Technology, Kermanshah 6715685420, Iran

**Keywords:** biomass sample, higher heating value, Elman neural network, topology tuning, training algorithm

## Abstract

The higher heating value (HHV) is the main property showing the energy amount of biomass samples. Several linear correlations based on either the proximate or the ultimate analysis have already been proposed for predicting biomass HHV. Since the HHV relationship with the proximate and ultimate analyses is not linear, nonlinear models might be a better alternative. Accordingly, this study employed the Elman recurrent neural network (ENN) to anticipate the HHV of different biomass samples from both the ultimate and proximate compositional analyses as the model inputs. The number of hidden neurons and the training algorithm were determined in such a way that the ENN model showed the highest prediction and generalization accuracy. The single hidden layer ENN with only four nodes, trained by the Levenberg–Marquardt algorithm, was identified as the most accurate model. The proposed ENN exhibited reliable prediction and generalization performance for estimating 532 experimental HHVs with a low mean absolute error of 0.67 and a mean square error of 0.96. In addition, the proposed ENN model provides a ground to clearly understand the dependency of the HHV on the fixed carbon, volatile matter, ash, carbon, hydrogen, nitrogen, oxygen, and sulfur content of biomass feedstocks.

## 1. Introduction

Since fossil fuels have been used extensively for energy [1], concerns have grown over the uncertain future of energy supplies as well as the adverse effects on the environment caused by their direct combustion [2,3]. Consequently, environmental protection is gaining much attention through the use of alternative energy sources [4,5,6]. As a renewable alternative to fossil fuels, energy production from biomass has gained considerable attention due to its considerable environmental advantages [7]. The thermochemical conversion of biomass is one of the most widely studied biofuel conversion technologies, but a strong focus on biomass large-scale production can result in controversies.

Thus, the use of waste-oriented biomass for energy generation as a sustainable and environmentally-acceptable method of generating energy can only be supported by using agricultural residues, municipal solid wastes, animal manure, sewage, and food waste [8,9].

A significant variation in the chemical and structural compositions of biomaterials used as feedstocks in thermochemical conversion results in a profound difference in the amount of energy that they contain. The energy content of biomass is lower than that of coal, so to achieve the same degree of thermal efficiency, more fuel is required [10]. It is difficult to standardize the product quality and process application due to the differences in the chemical and physical properties of biomass feedstocks [11]. Therefore, the applicability of biomass feedstocks in the context of energy conversion processes requires a variety of characterization and investigations [12]. Fuel heating values are generally reported in two ways: the lower (net) and the higher (gross) heating values [13].

In the selection and classification of feedstocks, the higher heating value (HHV) of a fuel is a crucial property [14]. The traditional method of measuring the HHV of a fuel sample is with an adiabatic oxygen bomb calorimeter. Liquid and solid fuel HHVs are determined using bomb calorimeters, but this technique is time-consuming and expensive [15]. Researchers can develop correlations to estimate fuel HHVs using the results of ultimate and/or proximate analyses [16]. While the ultimate technique necessitated a significant investment of both time and resources, the proximate analysis needed just a few straightforward procedures to identify the fuel mix’s constituents [16]. Proximity-based research is often more efficient and less expensive than other types of analysis. Proximate analysis, a technique for calculating the HHVs of fuels, has seen a recent surge in use [16]. Consequently, proximate analysis is more commonly used to predict the HHVs for fuels [17].

A wide range of empirical methodologies has been proposed to correlate the biomass HHV with the proximate and ultimate compositional information [18,19]. By separating cellulose, hemicellulose, lignin, and extractives from biomass, one can determine their relative amounts. In order to establish a relationship between HHV and biomass biochemistry, several equations have been developed [20,21]. Linear [22] and non-linear [23] equations are used extensively in the construction of these models. Because of the complexity of biomass, explaining how the proximate analysis data relates to the final analysis data for the HHV is challenging.

The literature includes several empirical correlations to predict the biomass HHV from ash (A), proximate analysis (PA), ultimate analysis (UA), volatile matter (VM), and fixed carbon (FC). For a variety of biomass, carbon (C), oxygen (O), hydrogen (H), sulfur (S), and nitrogen (N) are the four major components [13,16].

Ghugare et al. found that non-linear models might fare better than linear methods when estimating the biomass HHV [24]. Since PA/UA values fluctuate so often, it is challenging to develop a suitable non-linear empirical model without using computational models [24]. On the other hand, various machine learning models have extensively been tested to prove their trusted applications in various fields [25,26,27,28,29,30], including biotechnology [31,32]. The HHV of solid biomass was calculated using multilayer perceptron–artificial neural network (MLP–ANN) and genetic algorithm (GA) models by Ghugare et al. [24]. Thorough inquiries on the group were conducted. Hosseinpour et al. determined the HHV in biomass using an iterative neural network and a modified version of partial least squares [33]. By using the ANFIS model, Akkaya looked into the biomass’s heating value (HV) [34]. Intending to compare the performance of various ANN topologies, Uzun et al. devised a technique to calculate the HHV content of biomass. This was done so that the most effective topologies could be identified [35]. When combined with domain expertise and experimental data, the computational methods based on fuzzy inference systems can provide accurate models, as shown by Akkaya [34]. The calorific values of both individual and blends of biomass feedstocks may be predicted by ANN, as shown by Jakšić et al. [13]. Pattanayak et al. derived three ANN models to determine the HHV of different bamboo biomasses [36]. Each model was built using PA, UA, or a combination of the two. An improved artificial neural network model with particle swarm optimization was developed by Aladejare et al. to forecast the HHV of solid fuels like coal, lignite, and industrial waste, as well as biomasses like agricultural waste and forest waste [37]. This was done to calculate the HHV of coal, lignite, and other industrial waste biomasses (ANN–PSO). Statistical accuracy analysis showed that the ANN–PSO model was better than the multivariable regression correlation to predict the biomass HHV.

In this work, the Elman neural network (ENN), as a dynamic predictive tool with a great degree of flexibility and outstanding simulation performance, is used for the first time to estimate the biomass HHV. The structure-tuned ENN model can precisely anticipate the effect of the proximate and ultimate composition terms on the HHV and help find the best biomass type with the highest energy value.

## 2. Results and Discussions

### 2.1. Topology Tuning the ENN Model

**Figure 1:**
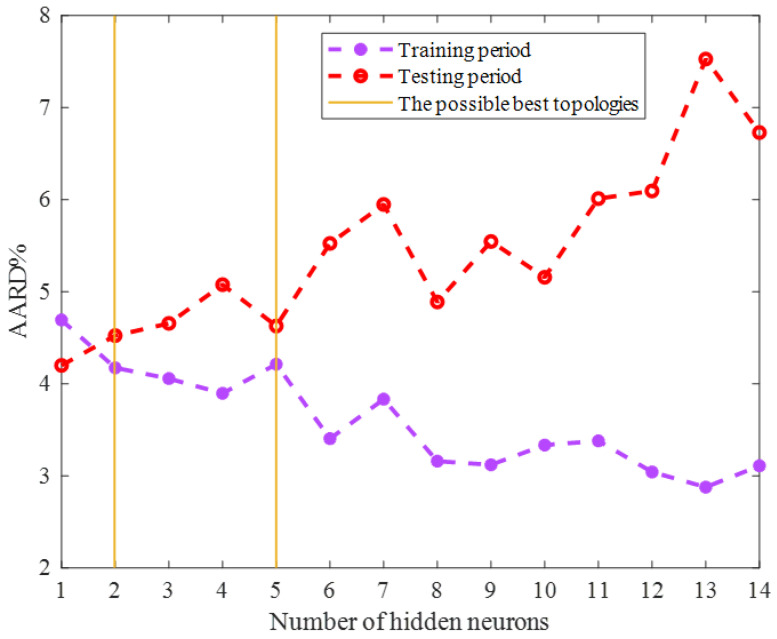
Sensitivity of the ENN–SCG accuracy on the number of hidden neurons.

**Figure 2:**
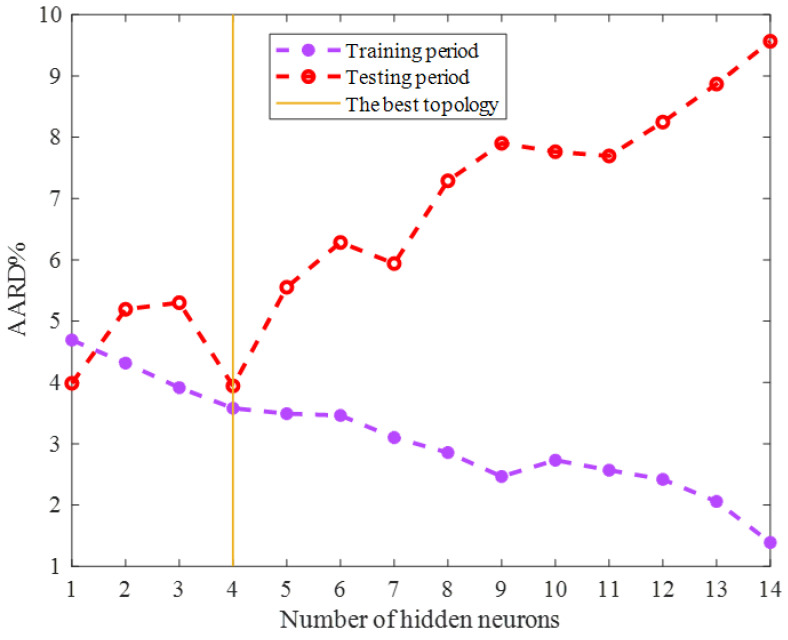
Sensitivity of the ENN–LM accuracy on the number of hidden neurons.

The optimization techniques are needed to find either the maximum or minimum value of an objective function [38,39]. This study employed two well-known optimization scenarios, i.e., the LM (Levenberg–Marquardt) [40] and SCG (Scaled Conjugate Gradient) algorithms, to adjust the ENN’s adjustable parameters. The accuracy of the ENN models with different sizes trained by the SCG and LM algorithms has been displayed in Figure 1 and Figure 2, respectively. This analysis approved that the ENN prediction accuracy is a function of the training scenario as well as the model size. Therefore, it was necessary to determine the most suitable ones by a comparative analysis.

Both Figure 1 and Figure 2 show that the model’s accuracy for predicting the training group increased by enlarging the ENN size (increasing the number of hidden neurons). On the other hand, the reliability of the ENN models for estimating the testing HHV samples decreased by enlarging the model size. Since the ENN model was necessary to estimate both the training and testing HHVs with acceptable accuracy, the best possible size of the ENN–SCG and ENN–LM models is also shown in Figure 1 and Figure 2. The ENN–SCG models with the two and five hidden neurons showed enough accuracy for predicting the training and testing datasets, while the ENN–LM only needed four hidden nodes to accurately estimate these two datasets.

Table 1 applies five statistical indices to compare the selected ENN models’ performance in the training and testing stages. This table also introduces the ENN models’ accuracy for predicting the whole HHV database.

The observed numerical indices approved that the ENN–LM model had a better performance than either the ENN–SCG 1 or the ENN–SCG 2 models. The absolute average relative deviation percent (AARD%), mean absolute error (MAE), relative absolute error percent (RAE%), and mean squared error (MSE) values achieved by the ENN–LM in the training and testing stages were smaller than those obtained from the ENN–SCG models. In addition, the correlation coefficient (R) values of the ENN–LM were higher than those obtained by the trained ENN model with the SCG algorithm.

**Figure 3:**
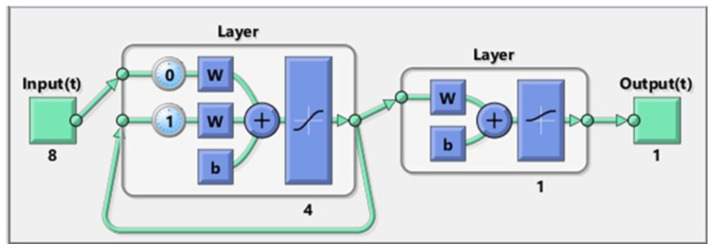
Topology of the developed ENN model for estimating the biomass HHV [41].

Hence, it can be claimed that the Levenberg–Marquardt had a better performance than the Scaled Conjugate gradient algorithm to accomplish the training phase of the Elman neural network. In addition, the LM algorithm provided the ENN model with a better generalization ability in the testing phase. Therefore, the ENN–LM model with only four hidden nodes (Figure 3) was selected as the best tool for predicting the biomass HHV.

It should be mentioned that all the developed ENN models have the logarithm sigmoid and hyperbolic tangent activation functions in the output and hidden layers, respectively. These functions help the ENN model to understand the nonlinear behavior of the biomass HHV in different operating conditions. Moreover, the continuous and differentiable characteristics of these activation functions are essential to adjust the ENN parameters by the training algorithm.

### 2.2. Performance Monitoring

This section relied on numerical and graphical investigations to evaluate how accurate the structure-tuned ENN–LM model was in predicting the biomass HHV.

#### 2.2.1. Training Stage

**Figure 4:**
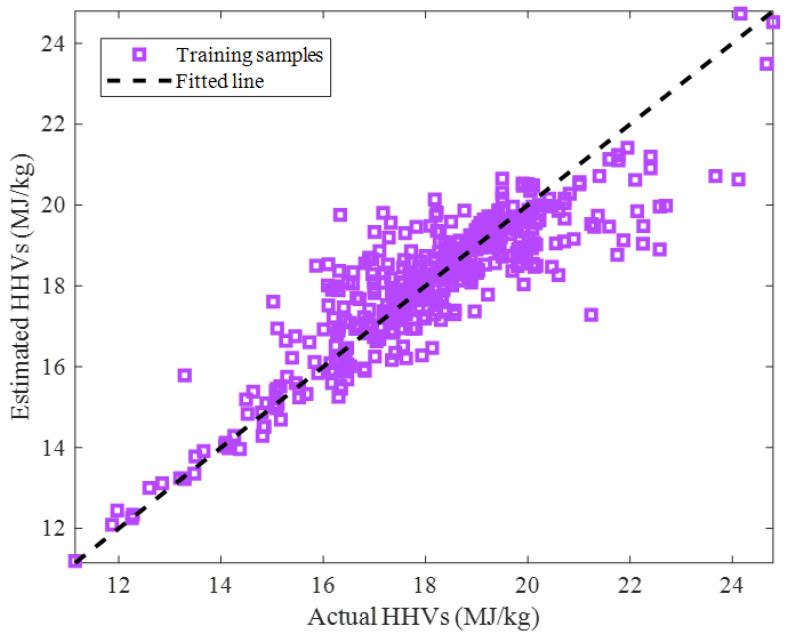
Predicted versus actual HHVs in the training step.

The cross-plot showing the actual versus calculated biomass HHVs for the training stage is depicted in Figure 4. The visual inspection of this figure and the observed R = 0.88335 indicate that an acceptable agreement existed between the actual and predicted biomass HHVs.

**Figure 5:**
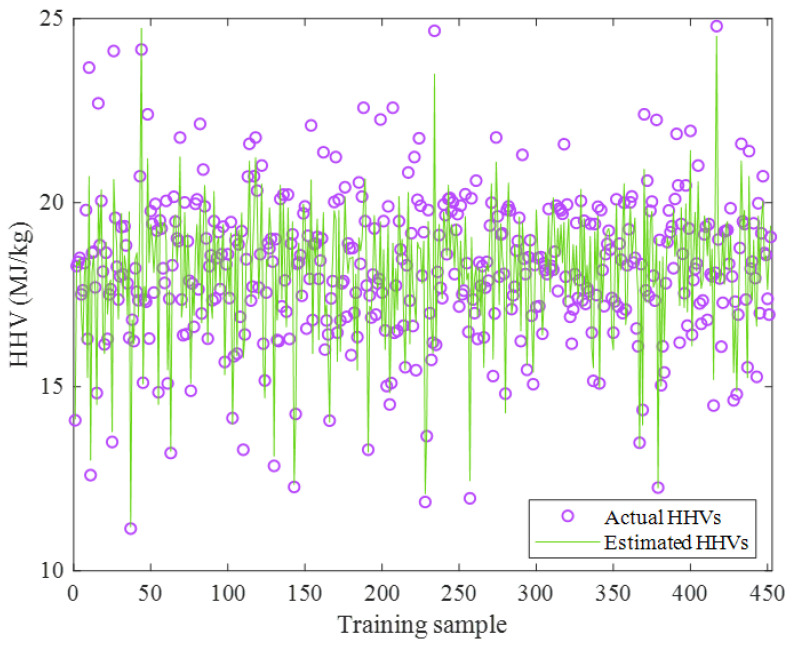
Actual HHV values and their counterpart estimations by the ENN–LM model.

The actual as well as predicted values of the biomass HHV shown in Figure 5 approved that the ENN–LM model was accurate enough in the training stage. The excellent performance of the ENN–LM model could be further justified by the numerical values of the statistical indices, i.e., AARD = 3.58%, MAE = 0.66, and MSE = 0.94.

**Figure 6:**
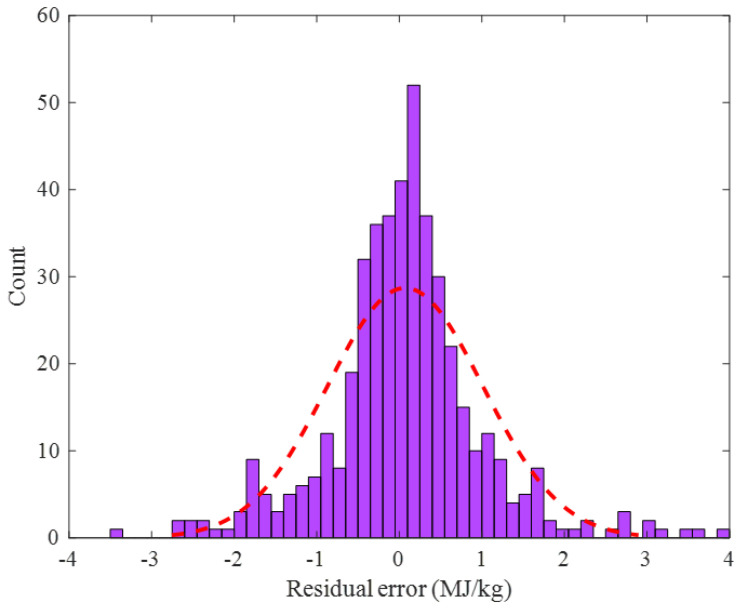
Frequency of the observed residual error of the ENN–LM model in the training stage.

The histogram of the residual error between the actual and the predicted values of the biomass HHV in the training step is shown in Figure 6. This figure approved that the training HHV samples had been predicted by the outstanding residual error ranging from −3.5 to 4 MJ/kg. Furthermore, the standard deviation and average values of these residual errors were very small, i.e., 0.942 and 0.071 MJ/kg, respectively.

#### 2.2.2. Testing Stage

**Figure 7:**
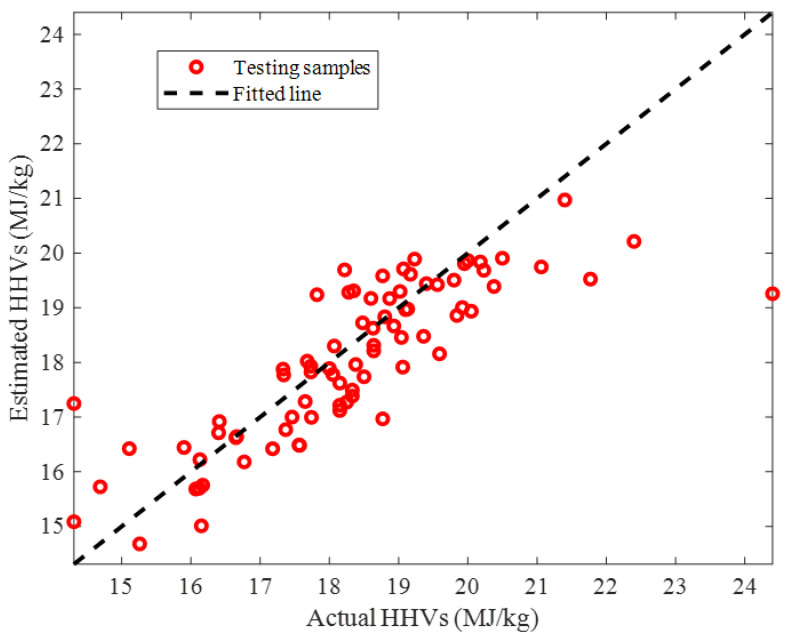
Estimated HHVs by the ENN–LM versus their counterpart actual values in the testing step.

The predicted HHVs by the ENN–LM model versus their counterpart actual values in the testing stage has been illustrated in Figure 7. The acceptable value of the coefficient of determination, i.e., R = 0.82255 showed that our small-size ENN–LM model was able to generalize its learning to the testing HHVs. The observed deviation between the actual and the predicted HHVs may be associated with the wide range of the involved biomass samples and their compositions, uncertainty in the experimental data, and model errors.

**Figure 8:**
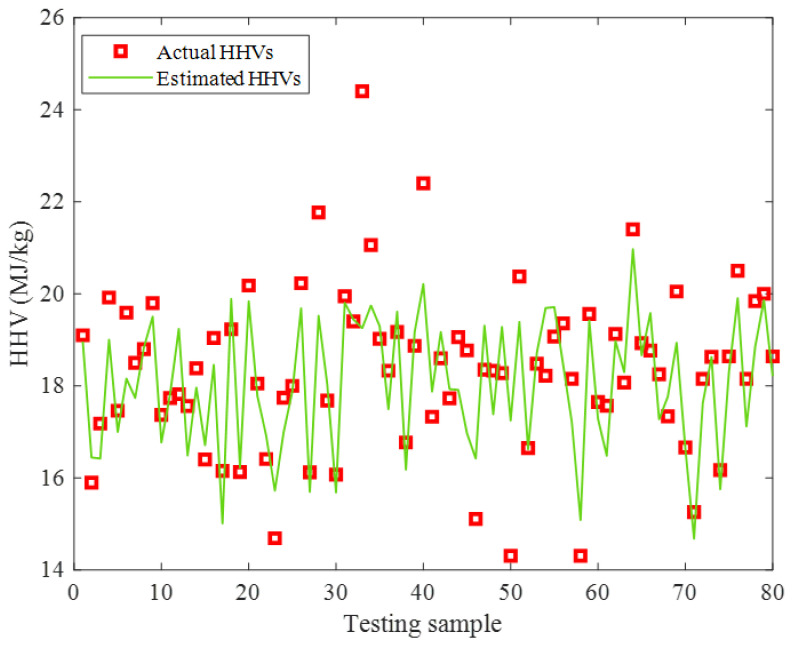
Actual HHVs and ENN–LM predictions in the testing step.

The actual HHVs and their related ENN–LM predictions in the testing stage have been simultaneously depicted in Figure 8. It can be seen that the proposed ENN–LM model reliably interpolated these highly scattered experimental HHVs. Moreover, the numerical values of the AARD = 3.94%, MAE = 0.73, and MSE = 1.03 approved the reasonable agreement between the actual and the predicted HHVs of the biomass feedstocks.

It can be simply seen that the constructed ENN–LM model underestimated five and overestimated four HHV samples in the testing stage. Since none of these HHVs were seen by the ENN–LM model before and they were highly scattered, this level of uncertainty is acceptable from the modeling perspective.

**Figure 9:**
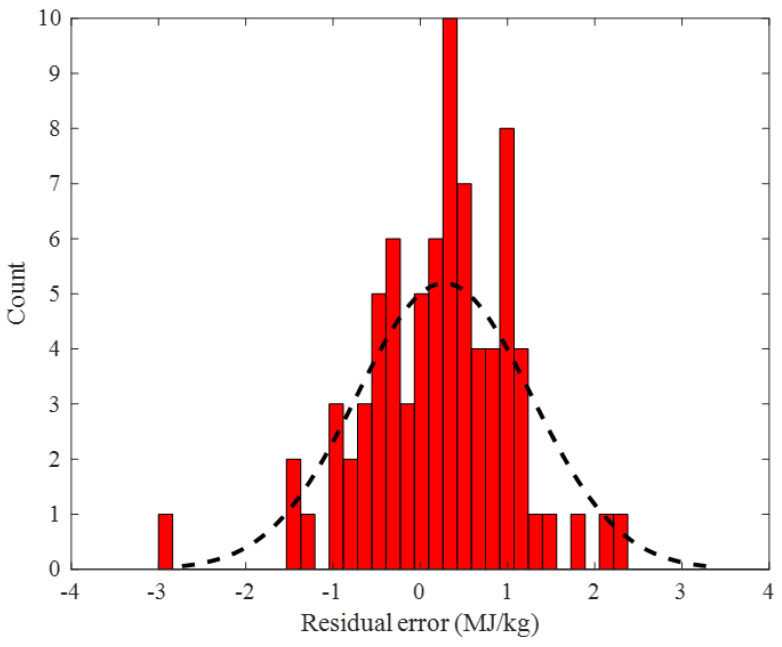
Frequency of the observed residual error of the ENN–LM model in the testing step.

Figure 9 indicates that the designed ENN–LM model predicted the testing samples of the biomass HHV with low residual errors ranging from −3 to 2.5 MJ/kg. The average and standard deviation of the testing stage residual errors were 0.274 and 1.00 MJ/kg, respectively. This figure also clarifies that a major part of the testing HHVs was estimated by the residual error of ~0 MJ/kg.

#### 2.2.3. Overall Data

**Figure 10:**
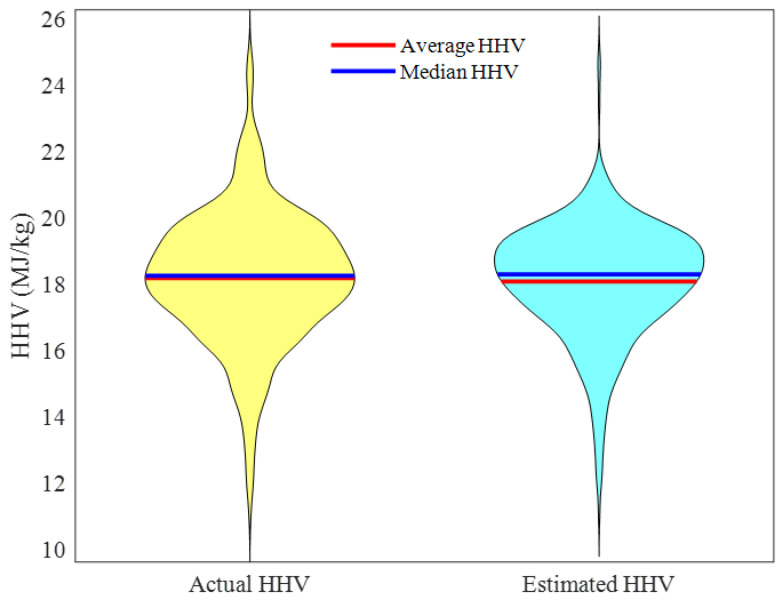
Violin graphs of the actual and predicted HHVs.

Figure 10 introduces the violin graph of the overall experimental HHVs and ENN–LM predictions. The complete similarity between these two graphs is an indicator of the acceptable accuracy of the structure-tune ENN–LM model for predicting the HHV of a wide range of biomass feedstocks.

Moreover, Table 2, which reports the experimental and predicted values of the median and average HHVs, approves an excellent performance of the proposed ENN–LM model. It can be seen that a slight deviation existed between the actual and modeling values.

## 3. Materials and Methods

### 3.1. Elman Neural Network

**Figure 11:**
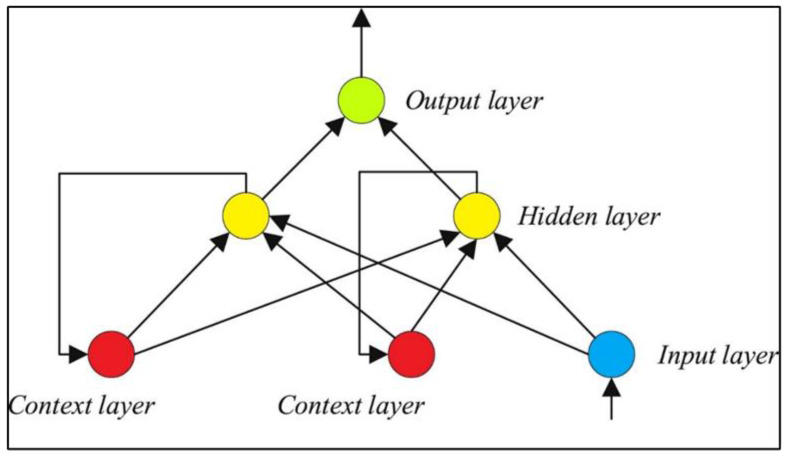
The general topology of a single hidden layer ENN model.

Based on Figure 11, the Elman neural network (also known as the recurrent neural network) is made up of the input, hidden, context, and output layers. Indeed, the ENN is a multilayer perceptron neural network with a feedback connection between the hidden and input layers [42]. As Figure 11 shows, the context layer gets its inputs from the hidden layer’s output. This internal feedback connection increases the network’s ability to process dynamic systems. ENNs possess short-term memory capabilities and are widely used as a means of managing either classification or approximation problems [42].

### 3.2. Data Collection

A model was developed to estimate the HHV of biomass data using eight independent variables, including FC, VM, ash contents on a dry basis, C, O, H, S (ash-free distribution), and N as inputs. Based on the input data (*X*_1_ to *X*_8_), the modeling method seeks to find *y* that best fits the data as follows:

(1) $$y\left(X_{1},\;X_{2},\;X_{3},\;X_{4},\;X_{5},\;X_{6},\;X_{7},\;X_{8}\right)$$

where *X*_1_, *X*_2_, *X*_3_, *X*_4_, *X*_5_, *X*_6_, *X*_7_, and *X*_8_ refer to the model inputs which are FC, VM, Ash, C, H, O, N, and S content of a biomass sample, and *y* denotes HHV.

The modeling procedure used 532 biomass data sets alongside their corresponding HHVs for use in the simulations. Table 3 presents the dependent/independent variables and their statistical information.

#### 3.2.1. Data Distribution in Model Development and Validation Stages

The researchers randomly took 452 out of 532 available data patterns to use in the training stage. In addition, the overtraining was monitored by the remaining 80 samples to use as test data. Consequently, 85% of the dataset was used for training, while 15% was chosen randomly for testing.

#### 3.2.2. Accuracy Evaluation

With the use of statistical testing, the most promising ideas were whittled down to a select few. Metrics like residual error (Equation (2)), standard deviation (Equation (3)), mean absolute error (Equation (4)), relative absolute error (Equation (5)), mean square error (Equation (6)), coefficient of determination (Equation (7)), and average absolute relative deviation (Equation (8)) abbreviated by *RE*, *SD*, *MAE*, *RAE*%, *MSE*, *R*, and *AARD*% were computed to assess the quality of the developed models. Each of these variables is defined in a different way using the following equations [43,44]:

(2) $$RE=\;HHV_{{}^{i}}^{act}-HHV_{i}^{est}\;\;\;\;\;\;\;i\;=\;1,\;2,\;\ldots ,\;n$$

(3) $$SD\;=\;\sqrt{\;\sum_{i=1}^{n}\left(1/n\right)\;\times {\left(RE_{i}\;-\;\bar{RE}\right)}^{2}}$$

(4) $$MAE=\;\left(1/n\right)\;\;\times \;{\sum_{i=1}^{n}\left|HHV^{act}-HHV^{est}\right|}_{i}$$

(5) $$RAE\%\;=\;100\;\times \;\sum_{i=1}^{n}{\left|HHV^{act}-HHV^{est}\right|}_{i}/\sum_{i=1}^{n}\left|HHV_{i}^{act}-\bar{HHV^{act}}\right|$$

(6) $$MSE=\;\left(1/n\right)\;\;\times \;{\sum_{i=1}^{n}\left(HHV^{act}-HHV^{est}\right)}_{i}^{2}$$

(7) $$R=\sqrt{1-\;\left\{\sum_{i=1}^{n}{\left(HHV^{act}-HHV^{est}\right)}_{i}^{2}/\sum_{i=1}^{n}{\left(HHV_{i}^{act}-\bar{HHV^{act}}\right)}^{2}\right\}}$$

(8) $$AARD\%=\left(100/n\right)\;\times \;\sum_{i=1}^{N}{\left|HHV^{act}-HHV^{est}\right|}_{i}/HHV_{i}^{act}$$

## 4. Conclusions

In this research, a systematic procedure was followed to construct the efficient Elman neural network model to anticipate the higher heating value of biomass feedstocks. The ENN topological features and its training algorithm are well-determined by a systematic procedure. The appropriate training algorithm and the optimum number of hidden neurons of the ENN have been determined by a combination of trial-and-error and sensitivity analysis. The proximate (fixed carbon, volatile matter, and ash) and the ultimate (carbon, oxygen, hydrogen, sulfur, and nitrogen) composition analyses of the biomass are the independent variables used to estimate the HHV. The results showed that the ENN with only four hidden nodes trained by the Levenberg–Marquardt algorithm should be introduced as the best tool for estimating the HHV of the biomass. This structure-tuned ENN predicted the HHV of 532 biomass samples with outstanding accuracy (i.e., MAE = 0.67, MSE = 0.96, and AARD = 3.63%). The perfect compatibility between the actual HHVs and their associated predicted values by the ENN was also approved by different graphical investigations, including cross-plot, violin graph, and residual error monitoring. Our reliable ENN model could be easily employed to choose a biomass feedstock with the highest HHV as a fuel source. Since the bomb calorimeter analysis is not always available, interested readers may conduct the HHV modeling by ignoring this type of information.

## Figures and Tables

**Table 1:** Measuring the accuracy of selected ENN models by different indices.

Model Name	Topology	Dataset	AARD%	MAE	RAE%	MSE	R
ENN–SCG 1	8-2-1	Training collection	4.17	0.76	51.61	1.09	0.83021
		Testing collection	4.52	0.83	54.99	1.22	0.82787
		The whole data	4.23	0.77	52.13	1.11	0.82914
ENN–SCG 2	8-5-1	Training collection	4.21	0.77	51.16	1.06	0.84745
		Testing collection	4.63	0.85	64.51	1.20	0.77340
		The whole data	4.28	0.78	52.84	1.08	0.83733
ENN–LM	8-4-1	Training collection	3.58	0.66	43.54	0.94	0.88335
		Testing collection	3.94	0.73	56.28	1.03	0.82255
		The whole data	3.63	0.67	45.19	0.96	0.87566

**Table 2:** Key information of the violin graphs of actual and predicted HHVs.

Index	Formula	Actual HHV	Estimated HHV
Median HHV	$Med\;=\;\left(HHV_{n/2}\;+HHV_{n/2+1}\;\right)/2$	18.28	18.32
Average HHV	$\bar{HHV}\;=\;\sum_{i=1}^{n}HHV_{i}/n$	18.21	18.11

**Table 3:** Ultimate/proximate composition analysis and HHV of the studied biomass.

Variable (Unit)	Analysis Type	Average	Standard Deviation	Minimum	Maximum	Observations
Fixed carbon (wt%)	Proximate	17.49	6.71	0.00	59.30	532
Volatile matter (wt%)		75.30	8.91	7.70	92.70	532
Ash (wt%)		6.21	6.98	0.10	67.10	532
C (wt%)	Ultimate	45.88	5.67	14.61	97.18	532
H (wt%)		5.88	0.99	0.41	11.55	532
O (wt%)		43.26	7.16	0.00	81.80	532
N (wt%)		1.04	1.07	0.00	6.75	532
S (wt%)		0.19	0.34	0.00	4.90	532
HHV (MJ/kg)	-	18.21	1.97	11.15	24.80	532

## Data Availability

All data is contained within the manuscript.
